# Effect of graphene substrate type on formation of Bi_2_Se_3_ nanoplates

**DOI:** 10.1038/s41598-019-41178-1

**Published:** 2019-03-18

**Authors:** Jana Andzane, Liga Britala, Edijs Kauranens, Aleksandrs Neciporenko, Margarita Baitimirova, Samuel Lara-Avila, Sergey Kubatkin, Mikhael Bechelany, Donats Erts

**Affiliations:** 1 Institute of Chemical Physics, University of Latvia, Riga, LV1586 Latvia; 2 Department of Microtechnology and Nanoscience, Chalmers University of Technology, SE-41296 Gothenburg, Sweden; 3 National Physical Laboratory, Hampton Road, Teddington, TW11 0LW UK; 4 European Institute of Membranes, University of Montpellier, CNRS, ENSCM, 34095 Montpellier, France

## Abstract

Knowledge of nucleation and further growth of Bi_2_Se_3_ nanoplates on different substrates is crucial for obtaining ultrathin nanostructures and films of this material by physical vapour deposition technique. In this work, Bi_2_Se_3_ nanoplates were deposited under the same experimental conditions on different types of graphene substrates (as-transferred and post-annealed chemical vapour deposition grown monolayer graphene, monolayer graphene grown on silicon carbide substrate). Dimensions of the nanoplates deposited on graphene substrates were compared with the dimensions of the nanoplates deposited on mechanically exfoliated mica and highly ordered pyrolytic graphite flakes used as reference substrates. The influence of different graphene substrates on nucleation and further lateral and vertical growth of the Bi_2_Se_3_ nanoplates is analysed. Possibility to obtain ultrathin Bi_2_Se_3_ thin films on these substrates is evaluated. Between the substrates considered in this work, graphene grown on silicon carbide is found to be the most promising substrate for obtaining of 1–5 nm thick Bi_2_Se_3_ films.

## Introduction

Recently, the integration of graphene with other Dirac materials (for example, topological insulator bismuth selenide (Bi_2_Se_3_)) has gained interest due to its potential to be used as a platform for the experimental realization of new room-temperature applications 2D TI structures^1,2^. For instance, these include thermoelectric devices^3^ with Bi_2_Se_3_ being one of the best thermoelectric materials for near-room temperature applications. Other important applications are flexible composites for the use as anodes in lithium-ion batteries^4^ and active elements in high-performance optoelectronic devices^5^.

Bi_2_Se_3_ has rhombohedral crystal structure in the space group $R\bar{3}m$ with weak Van der Waals forces along the c-axis^6^. Each charge neutralized layer (quintuple layer, Q-layer) is formed by five covalently bonded atomic sheets (Se-Bi-Se-Bi-Se). Bi_2_Se_3_ can be deposited on solid substrates by variety of methods, including molecular beam epitaxy (MBE)^2,7^, physical and chemical vapour deposition (PVD and CVD)^3,5,8^, thermal evaporation^9^.

Recent theoretical calculations performed for thermoelectrical properties of thin bismuth chalcogenide films predict that the reduction of the thickness of thin film below 10 nm may result in an increase of the existing thermoelectric figure of merit of this thin film by an order of magnitude^10^. Deep knowledge of Bi_2_Se_3_ nucleation and growth on various substrates is crucial for obtaining ultrathin continuous films of this material.

The PVD method remains one of the most promising to be used at industrial scale as it is simple, cost-effective and allows for large-scale production. However, unlike the highly controllable MBE method, growth of ultrathin Bi_2_Se_3_ nanostructures or continuous films by PVD deposition on different surfaces remains challenging. Hampering of lateral growth of the nuclei by physical and chemical defects of the substrate surface prevents layer-by-layer growth of Bi_2_Se_3_ films^11^. For obtaining of ultrathin nanostructures or films, atomically-flat, chemically-inert substrates are favourable.

Mica and graphene are recognized as excellent substrates for Bi_2_Se_3_ growth. Mica is chemically inert substrate with atomically flat surface ideal for Van der Waals epitaxy of Bi_2_Se_3_^1,12–16^. Despite a significant lattice mismatch between the mica and Bi_2_Se_3_ (~25%), the reported thicknesses for Bi_2_Se_3_ nanoplates deposited by PVD technique on synthetic fluorophlogopite mica surface are as small as 2–3 nm^15^. PVD synthesis on natural muscovite mica, which is cheaper alternative to synthetic mica, results in formation of Bi_2_Se_3_ nanoplates with thicknesses down to approx.10 nm. Continuous Bi_2_Se_3_ thin films grown on mica have thicknesses starting from 50 nm^12,16^. The thermoelectric power factor of these thin films is comparable with the Bi_2_Se_3_ thin films obtained by MBE techniques^12^. As for graphene, a very small lattice mismatch between graphene and Bi_2_Se_3_ (~2.9%)^1^ makes it very attractive substrate to promote epitaxial growth of Bi_2_Se_3_ for the applications, where electrically conductive substrate is required.

Graphene substrates can be prepared by different methods, including CVD synthesis of graphene followed by its polymer-assisted transfer onto the desired substrate, mechanical exfoliation of highly ordered pyrolytic graphite (HOPG), thermal decomposition of SiC substrates (G/SiC), and others^17^. All these methods have advantages and drawbacks in terms of controllability, graphene quality, and costs. It is important to clarify how graphene substrates prepared by different techniques may differently affect the nucleation, further growth and properties of deposited on these substrates Bi_2_Se_3_.

This work is focused on a study of growth dynamics of Bi_2_Se_3_ nanoplates deposited by catalyst-free PVD technique on prepared by different methods (CVD followed by polymer-assisted transfer with and without post-annealing, SiC thermal decomposition) graphene substrates. The dimensions of deposited under the same experimental conditions onto graphene substrates nanoplates are compared with the dimensions of the Bi_2_Se_3_ nanoplates deposited on mica and exfoliated multilayer HOPG flakes used as reference substrates. Factors affecting nucleation and growth of the nanoplates are discussed, and the possibilities for further obtaining of ultrathin Bi_2_Se_3_ films on graphene substrates are evaluated.

## Results and Discussion

**Figure 1:**
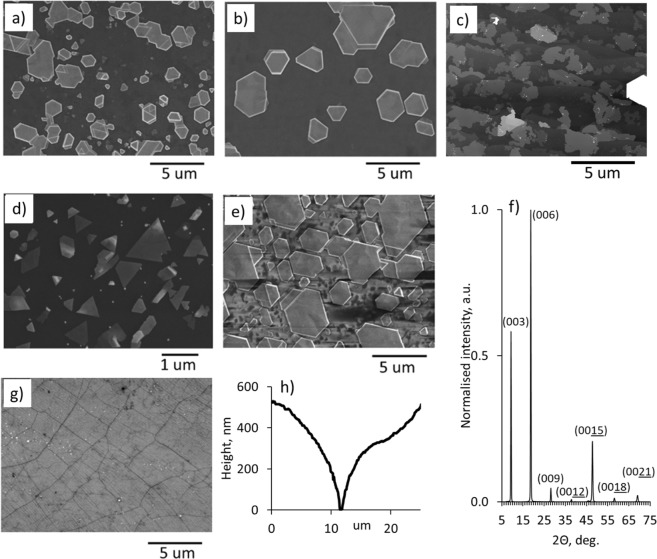
Scanning electron microscope (SEM) images of Bi_2_Se_3_ nanoplates deposited on the surface of (**a**) as-transferred CVD graphene; (**b**) post-annealed CVD graphene; (**c**) G/SiC substrate; (**d**) multilayer HOPG flake; (**e**) mica; (**f**) illustrative XRD pattern of a Bi_2_Se_3_ thin film formed from the nanoplates on the surface of as-transferred CVD graphene; (**g**) SEM image of CVD grown graphene transferred onto a glass substrate; (**h**) height profile of a typical grain boundary on the surface of copper substrate used for the CVD graphene synthesis.

Figure 1a–e show illustrative examples of Bi_2_Se_3_ nanoplates deposited on as-transferred and post-annealed CVD graphene, G/SiC, exfoliated multilayer HOPG flake and mica substrates.

Due to the intrinsically anisotropic bonding nature^18^, normally the most energetically favourable orientation of Bi_2_Se_3_ nanoplates is with c-axis oriented perpendicularly (i.e. with Q-layers parallel to the surface of the substrate) to the commonly used for Bi_2_Se_3_ deposition substrates as graphene^3,5,7–9^, SiO_2_^11–13^ and mica^14,15^. Diffraction peaks of XRD patterns obtained for the continuous Bi_2_Se_3_ thin films formed from the coalesced nanoplates on as-transferred CVD graphene surface (Fig. 1f) can be indexed to the rhombohedral structure with cell units of a = b = 4.139 Å and c = 28.636 Å^19^ (ref. code 00-033-0124), and are affiliated to a group (0 0 3n). This proves the growth of the Bi_2_Se_3_ layers in a single-crystalline phase with c-axis oriented perpendicularly to the substrate.

**Figure 2:**
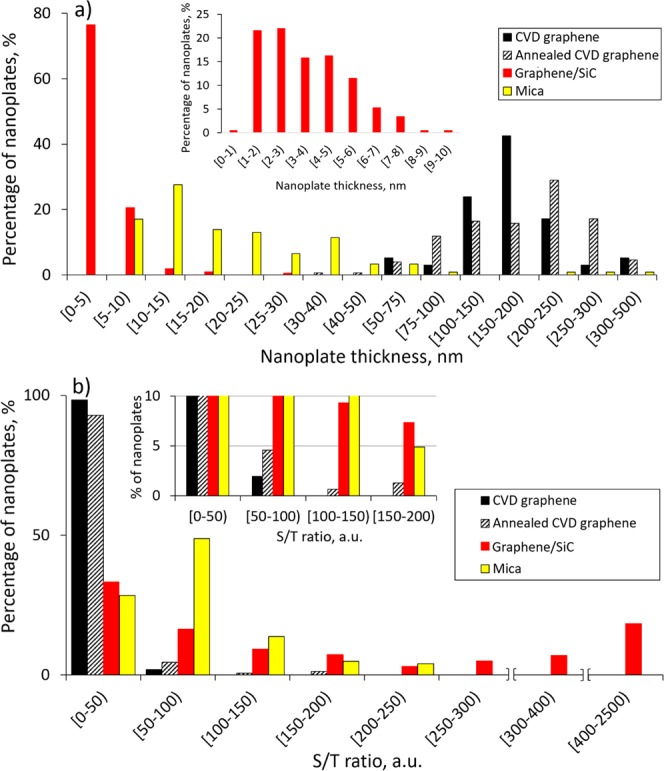
Comparative histograms: (**a**) of the thicknesses of Bi_2_Se_3_ nanoplates, deposited on different graphene and mica substrates. Inset – histogram of the thicknesses of Bi_2_Se_3_ islands deposited on G/SiC substrates for the thickness range 1–10 nm; (**b**) of the S/T parameter of Bi_2_Se_3_ nanoplates, deposited on different graphene substrates and mica. Inset – closer look at the spread of the S/T parameter of the deposited on different substrates Bi_2_Se_3_ nanoplates.

**Figure 3:**
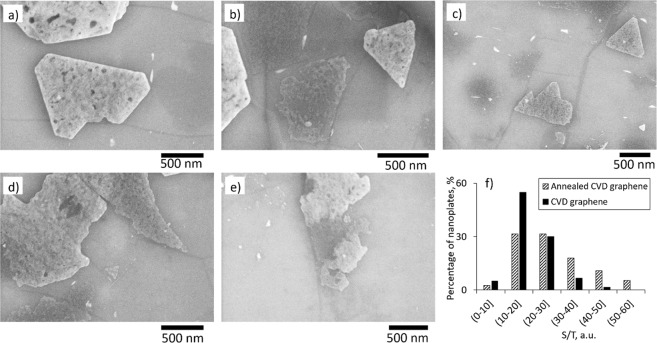
(**a**–**e**) SEM images of the initial stages of Bi_2_Se_3_ nanoplates formation on CVD graphene surface; (**f**) comparative histogram of the S/T ratios of the initial formation stage of the nanoplates on as-transferred and post-annealed CVD graphene.

Generally, the top surfaces of the Bi_2_Se_3_ nanoplates are Se-passivated^20^, resulting in much faster lateral growth of nanoplates than vertical due to diffusion of Bi and Se adatoms to the edges of the nanoplates. However, it is found in this study that the ranges of thicknesses and surface areas of the deposited under the same synthesis conditions nanoplates differ greatly for different graphene substrates. For relative comparison nanoplates dimensions, a parameter “surface-to-thickness ratio” (S/T) is used. For the S/T calculation, the surface area of the upper facet of a nanoplate is divided by the averaged thickness of this nanoplate. Both surface area and thickness are determined by the analysis of AFM scans of the nanoplates. The domination of the lateral growth direction over the vertical is indicated by higher values of S/T parameter. Data on the ranges of nanoplates thicknesses, surface areas and S/T ratios are summarised in Table 1 and comparative histograms in Figs 2 and 3f.

**Table 1:** Dimensions of Bi_2_Se_3_ nanoplates deposited on different types of CVD graphene substrates, G/SiC, multilayer HOPG flake, and mica substrates.

Substrate	Thickness of Bi_2_Se_3_ nanoplates T, nm	Bi_2_Se_3_ nanoplates surface area S, um^2^	Surface-to-thickness ratio S/T, x10^3^ a.u.
CVD grapheme transferred on a glass substrate	50–375	0.4–11	2–60
Annealed CVD grapheme transferred on a glass substrate	30–360	0.4–30	2–170
Graphene on SiC substrate	1–20	1–50	30–2500
Exfoliated HOPG flake transferred on a glass substrate	18	1.13	63
	20	0.5	25
	21	1.85	88
	22	1.1	49
	22	1.4	63
	27	0.43	16
Natural mica (reference substrate)	6–240	0.3–20	11–240

Thicknesses of the 75% of Bi_2_Se_3_ nanoplates deposited on mica (considered as a reference substrate) are below 25 nm, and 17% have thicknesses between 6 and 10 nm (Fig. 2a). It was expected that in contrast with mica, showing lattice mismatch with graphene of approx. 25%, a very small lattice mismatch of 2.9% between Bi_2_Se_3_ and a pure defect-less graphene will promote strain-free epitaxial 2D growth of crystalline Bi_2_Se_3_ layers^21,22^. At the initial stages of the Bi_2_Se_3_ deposition on graphene surface, such growth would result in formation of thin and large 2D islands of Bi_2_Se_3_ with higher S/T ratios than of the nanoplates deposited on mica.

### As-transferred and post-annealed CVD graphene substrates

Unexpectedly, the minimal thicknesses of the Bi_2_Se_3_ nanoplates deposited on CVD graphene exceed the minimal thicknesses of the Bi_2_Se_3_ nanoplates deposited on mica by 5–9 times (Fig. 2a). The thicknesses of the Bi_2_Se_3_ nanoplates deposited on as-transferred CVD graphene vary from 50 nm up to 375 nm (Table 1), with 9% of the nanoplates being below 100 nm thick (Fig. 2a). In turn, the thicknesses of the nanoplates deposited on post-annealed CVD graphene vary from 30 nm up to 360 nm with 17% of them being below 100 nm. This allows to conclude that post-annealing of the CVD graphene results in slight reduction of the nanoplates thicknesses in comparison with the nanoplates deposited on as-transferred CVD graphene. However, the post-annealing of transferred CVD graphene does not allow to reduce the thicknesses of the deposited nanoplates down to the dimensions comparable with the nanoplates deposited on mica substrate.

Even though the most thicknesses of the Bi_2_Se_3_ nanoplates deposited on as-transferred and post-annealed CVD graphene substrates fall in nearly the same range 50–300 nm (Table 1, Fig. 2a), the number of the nanoplates per unit area and lateral dimensions of the nanoplates deposited on these two types of substrates differ. The number of the nanoplates per unit area for as-transferred CVD graphene is higher, while their lateral dimensions are noticeably smaller in comparison with the nanoplates deposited on post-annealed CVD graphene (Fig. 1a,b, Table 1). As it can be seen from the histogram (Fig. 2b) and Table 1, the S/T parameters of the nanoplates deposited on mica cover the range from 11 to 240 with ~30% in the range [0–50), ~50% in the range [50–100) and ~19% in the range [100–200)). In contrast, 98% of the S/T parameters of the nanoplates deposited on as-transferred graphene are in the range [0–50) and the other 2% are in the range [50–100).

The nanoplates deposited on post-annealed graphene have higher than for as-transferred CVD graphene S/T values spread in the wider range (Figs 2b and 3f). These S/T values overlap with the S/T values of the nanoplates on mica in the range 11–170 (Table 1, Fig. 2b inset). However, the percentage of the nanoplates with higher S/T ratios grown on post-annealed is significantly smaller than for the nanoplates grown on mica. This indicate that the surface of post-annealed CVD graphene promotes lateral growth of the Bi_2_Se_3_ nanoplates better than the surface of as-transferred CVD graphene, but not good enough for formation of ultrathin Bi_2_Se_3_ films.

Presumably, the thicknesses and number per unit area of the Bi_2_Se_3_ nanoplates are defined by the number of surface defects of CVD graphene. One of the main causes of the surface defects of CVD graphene is its domain structure, arising from the grainy structure of copper substrate used during the CVD deposition^23^. Figure 1g,h illustrate respectively scanning electron microscope (SEM) image of a CVD graphene and a height profile of a typical grain boundary on the surface of copper foil used for CVD graphene deposition. Since the edges of the copper domains are concave, it is possible that the graphene domains do not grow together completely. When transferring CVD graphene from the copper substrate, the domains may be joined mechanically. This may result in a large number of defects, creating potential barriers of 0.02–0.1V^13,24^, as well as in some number of dangling bonds alone the domain boundaries. Presumably, such domain boundaries may serve as obstacles for the nanoplates lateral growth, and at the same time as nucleation centres. The role of graphene domain boundaries in the formation of Bi_2_Se_3_ nanoplates is illustrated by the Fig. 3a–e, showing the initial stages of the nanoplates formation, occurring at high substrate temperatures (see the Methods section). The graphene domain boundaries interrupt symmetrical lateral growth of the nanoplates (for example see Fig. 3a), but at the same time may serve as nucleation centres (for example see Fig. 3d). The rugged surfaces of the nanoplates are due to the excess of Bi at the initial stages of the deposition, which is compensated during the later stages of the deposition occurring during the cooldown process (see the Methods section).

In addition to the impurities introduced by the domain boundaries, the CVD graphene may have significant number chemical surface defects formed during the transfer process^25^. For instance, it may be residual contamination with polymers used for the transfer. These defects also may serve as nucleation centres or lateral growth limiting factors for the nanoplates. Nucleation occurring on these defects together with hampering of the lateral growth of the nanoplates without affecting the speed of their vertical growth results in relatively high thicknesses of the nanoplates and their dense location. That means that the thin films grown on as-transferred CVD graphene will consist of higher number of grown together nanoplates per unit area in comparison with thin films grown on post-annealed CVD graphene. As the boundaries between the grains of nanostructures forming thin films are known to affect their electrical^26^ and thermal^27^ properties, the thermoelectric properties of the Bi_2_Se_3_ thin films deposited on as-transferred and post-annealed CVD graphene may differ.

Post-annealing of as-transferred CVD graphene leads to the partial healing of the chemical surface defects on the graphene surface^28^ and elimination of the polymer residues^29,30^. This reduces the number of possible nucleation centres that may also hamper the nanoplates lateral growth on the graphene surface. This results in an increase of lateral dimensions of the nanoplates and decrease of their location density. However, post-annealing does not completely remove the obstacles for Bi_2_Se_3_ nanoplates lateral growth on the CVD graphene surface, as the minimal thickness of the nanoplates decreases insignificantly (30 nm, Table 1) and does not achieve the average thickness of the Bi_2_Se_3_ nanoplates deposited on mica (~22 nm with minimal thicknesses of 6 nm). Most probably, this effect can be attributed to the influence of the large domain boundaries, formed during the graphene growth on the copper substrates and remaining on the CVD graphene surface after its post-annealing. It should be noted that the nanoplates with thicknesses below 10 nm and large S/T parameters are crucial for formation of ultrathin continuous Bi_2_Se_3_ thin films. The conclusion is that both as-transferred and post-annealed CVD graphene substrates may be useful for obtaining of thick (~150–300 nm) nanostructured Bi_2_Se_3_ films. For obtaining of ultrathin Bi_2_Se_3_ films, alternative to CVD graphene substrates as, for instance, G/SiC should be considered.

### Graphene/SiC and exfoliated HOPG flakes

Using of G/SiC substrates allows to avoid the influence of such surface defects as domain boundaries or transfer-caused defects (polymer residues etc), increasing the number of nucleation centres and hampering the lateral growth of the Bi_2_Se_3_ nanoplates.

Thicknesses of the 75% of Bi_2_Se_3_ islands formed on the surface of G/SiC are found to be below 5 nm (Table 1, Fig. 2a). Thicknesses of nearly half (43%) of the Bi_2_Se_3_ islands are having thicknesses 3 nm and below (Fig. 2a inset). At average, thicknesses of the Bi_2_Se_3_ islands deposited on G/SiC substrates are ~5, 10 and 15 times smaller than the minimal thicknesses of the Bi_2_Se_3_ nanoplates deposited on as-transferred CVD graphene, post-annealed CVD graphene and mica substrates respectively.

**Figure 4:**
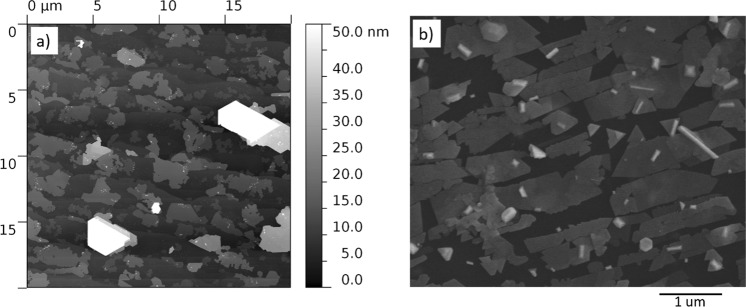
(**a**) Bi_2_Se_3_ islands formed on G/SiC substrate; (**b**) SEM images of the Bi_2_Se_3_ nanoplates formed on an exfoliated HOPG flake.

It is observed that Bi_2_Se_3_ nucleation starts at the jagged edges of G/SiC terraces formed during the thermal decomposition of the SiC substrate (Fig. 4a). The lateral growth of Bi_2_Se_3_ 2D islands seems to be limited by the edges of the terraces similarly to the previously reported MBE deposition of Bi_2_Se_3_ on G/SiC substrates^7^.

Presumably, the terraces formed on the G/SiC substrates are the main factor influencing Bi_2_Se_3_ nucleation and lateral growth due to the presence of the double-layer graphene areas along their edges. As it was reported in^31,32^, this double-layer graphene exhibits higher surface energy in comparison with the single-layer graphene. Possibly, the Bi_2_Se_3_ nucleation may start at the promoting deposition areas of double-layer graphene and continue epitaxially.

In contrast with the S/T parameters of Bi_2_Se_3_ nanoplates deposited on as-transferred CVD graphene (96% of S/T is below 30) and post-annealed CVD graphene (81% of S/T is below 30), the S/T parameters for the Bi_2_Se_3_ islands formed on the G/SiC substrates are starting from 30 and are spread up to 2500 (Table 1, Fig. 2b). For the comparison, the amount of the deposited-on mica Bi_2_Se_3_ nanoplates with S/T below 30 is just about 7%. Thus, the S/T parameters for the Bi_2_Se_3_ islands deposited on G/SiC cover almost all the values of the S/T parameters for the nanoplates deposited on mica and expand up to the significantly (up to an order of magnitude) higher S/T values. 45% of the Bi_2_Se_3_ islands on G/SiC substrates are having higher S/T parameters than the largest S/T of mica-supported nanoplates (Fig. 2b). The high S/T ratios at small average thickness of the nanostructures are vital for formation of ultrathin films. This means that between the useful for practical application graphene substrates, the epitaxial graphene grown on SiC surface is the best substrate for 2D growth of Bi_2_Se_3_. In perspective, G/SiC substrates could be used for obtaining of ultrathin Bi_2_Se_3_ thin films with thicknesses 1–5 Q-layers. Also, separate Bi_2_Se_3_ nanoplates with large (up to 50 um^2^) surface areas, thicknesses up to 20 nm (Table 1) and smooth graphene/Bi_2_Se_3_ interface can be formed on these substrates.

For the comparison with G/SiC substrates and illustration of the role of terraces in formation of Bi_2_Se_3_ nanoplates, the exfoliated multilayer HOPG flakes are used. As it is well known, graphene obtained by mechanical exfoliation of HOPG is of the best quality. However, the number of graphene layers in an exfoliated flake is difficult to control. Also, the HOPG flakes have terraced structure formed during the mechanical exfoliation. Average thickness of the nanoplates obtained on the flakes of exfoliated HOPG is ~21 nm with S/T ratios 18–88 (Table 1). These parameters are comparable with the parameters of the Bi_2_Se_3_ nanoplates deposited on mica. Characteristic for the Bi_2_Se_3_ triangular shapes of the separate nanoplates (Fig. 1d) indicate similar to mica absence of minor surface defects of this type of substrate. However, the Bi_2_Se_3_ nanostructures deposited on the HOPG flake surface are lined up, presumably along the graphite terrace edges. Such lining-up is clearly seen in the areas of the HOPG flake with more dense location of the Bi_2_Se_3_ nanoplates (Fig. 4b). This allows to conclude that in the case of HOPG flake the growth mechanism is partly similar to this on the G/SiC substrate. The nucleation of the nanoplates starts at the edges of the terraces and expands epitaxially along the terrace surface. The higher average thickness of the nanoplates deposited on the exfoliated HOPG flake in comparison with the average thickness of the nanoplates deposited on G/SiC may possibly be explained by the specific features of G/SiC substrates as the presence of double-layer graphene with higher surface energy along the edges of the terraces and strain-less surface of the graphene, effectively promoting nucleation and epitaxial growth of the Bi_2_Se_3_.

## Summary

Nucleation and further growth of Bi_2_Se_3_ nanoplates deposited by catalyst-free PVD method under the same experimental conditions on prepared by different methods graphene substrates is studied. The dimensions of these nanoplates are analysed and compared with the dimensions of Bi_2_Se_3_ nanoplates deposited on mechanically exfoliated natural mica and multilayer HOPG flake as reference substrates.

Following the analysis of the nanoplates dimensions in relation to specific features of the substrate, it can be concluded that the lateral growth of the nanoplates on as-transferred CVD graphene may be slowed down by the potential barriers of its domain boundaries, as well as by the other surface defects arising from the CVD graphene growth and transfer process. Hampering of the lateral growth of the nanoplates while vertical growth rate remains the same results in minimal thicknesses of the Bi_2_Se_3_ nanoplates of 50 nm.

Post-annealing of the CVD graphene eliminates the polymer contamination and heals minor graphene surface defects but is not affecting the graphene domain boundaries. Elimination of minor defects of the CVD graphene surface results in reduction of minimal thicknesses of the deposited nanoplates down to 30 nm and an increase of their S/T parameters in comparison with the as-transferred CVD graphene. Thin films grown on as-transferred CVD graphene are expected to have higher number of the nanoplates per unit area in comparison with thin films grown on post-annealed graphene. The difference in the number of the nanoplates per unit area may affect the electrical and thermal properties of the Bi_2_Se_3_ thin films grown on as-transferred and post-annealed CVD graphene substrates.

Graphene grown directly on the SiC substrate by its thermal decomposition method is found to be a very good substrate for 2D growth of Bi_2_Se_3_. The Bi_2_Se_3_ nucleation starts at the edges of the terraces of G/SiC substrate (presumably covered with double-layer graphene) and continues epitaxially. Lateral growth of Bi_2_Se_3_ islands is limited by the edges of the terraces. Similar growth mechanism is observed on the mechanically exfoliated multilayer HOPG flakes, where the deposited Bi_2_Se_3_ nanoplates were lined-up along the graphite terraces. However, the average thickness of the nanoplates deposited on HOPG flakes (~21 nm) is by at least 5 times larger than the average thickness of the nanoplates deposited on G/SiC (~4 nm). Possibly, this may be explained by specific issues of the G/SiC substrates as double-layer graphene with higher surface energy along the edges of the terraces and strain-less graphene surface. Thus, the G/SiC substrates are found to be the most perspective for obtaining of Bi_2_Se_3_ thin films with thicknesses below 5 nm. However, the drawback of using the G/SiC substrates is limitation by the wafer sizes (currently 6” available) and costs.

## Methods

### Bi_2_Se_3_ nanoplates synthesis

Bi_2_Se_3_ nanostructures were synthesized by modified catalyst-free vapour-solid deposition method reported elsewhere^3,33–35^ using a quartz tube furnace (GCL-1100X, MTI Corp). The source material (Bi_2_Se_3_, 99.999%, Sigma Aldrich) was placed in a quartz boat in the centre of the furnace tube, while the substrate was located downstream relative to the source material.

For the synthesis, the furnace tube was flushed with N_2_, pumped down to the base pressure 5 Torr and sealed. The centre of the quartz tube was heated at a rate of 13 °C/min to 585 °C. The substrate temperature for Bi_2_Se_3_ deposition was 410–450 °C. The temperature was held constant (at 585 °C) for 15 minutes for the initial formation of the nanoplates. Than the furnace was naturally cooled down to 465 °C. During this cooling process, the further growth of the nanoplates together with their enrichment with Se occurs. At furnace temperature of 465 °C, the furnace tube was filled with N_2_ to atmospheric pressure, sealed and cooled down to room temperature. For the study of the initial formation stages of the nanoplates, the substrate was taken out from the furnace tube at 585 °C and rapidly cooled down under the N_2_ gas flow.

### Substrate preparation

Glass substrates were washed in 2-propanol and distilled water, followed by drying under N_2_ flow. Muscovite mica sheets (Agar Scientific) were mechanically cleaved immediately before the synthesis process.

#### As-transferred CVD graphene

Monolayer graphene was prepared by chemical vapour deposition method on copper foil and transferred on the quartz/glass substrates using polymer-assisted technique described elsewhere^20^.

#### Annealing of CVD graphene

Glass substrates with transferred by polymer-assisted technique graphene was annealed consequently in air (250 °C, ambient pressure, 60 min) and H_2_/Ar flow (0.2/0.4 L/min, 250 °C, base pressure 0.5 Torr, 60 mins).

#### Exfoliation of HOPG

HOP graphite flakes were mechanically exfoliated from the macro piece of HOPG and transferred onto the pre-cleaned glass substrate using adhesive tape (3M^TM^ 8805).

#### G/SiC substrates

Monolayer graphene was grown on the Si-face of SiC using thermal decomposition of 7 × 7 mm SiC substrates (Cree, Inc). The 3” SiC wafer is diced into 7 × 7 mm chips, which are cleaned using solvents (acetone, isopropyl alcohol) and blow-dried with N_2_. Chips are then cleaned using RCA 1 and 2 processes and dipped in HF (3%) for 30 seconds. The samples are loaded in a reactor for epitaxial graphene growth equipped with an inductive heater. Graphene growth takes place in argon atmosphere (P = 600 Torr) at a temperature of 1700 °C for 5 min. After growth, optical microscopy reveals a typical surface coverage of >90% monolayer graphene.

### Investigation methods

Morphology, thickness, structure and composition of obtained Bi_2_Se_3_ nanostructures and nanostructured layers were inspected using field emission scanning electron microscope (SEM) Hitachi S-4800 equipped with an energy-dispersive X-ray (EDX) analyser Bruker XFLASH 5010 and atomic force microscopes Asylum Research MFP-3D and Bruker Dimension ICON.

For the statistical analysis, 5 to 9 AFM scans of size 20 × 20 μm each were obtained at different locations on the sample. The number of measured nanoplates was 110–150 for each type of the substrate with an exclusion of exfoliated graphene substrate. Due to the technical difficulties, 6 nanoplates were measured on the surface of exfoliated graphene.

XRD characterization of the thin films was performed by a Philips X’Pert MRD with a Cu Kα radiation source.
